# Disorder of extreme stress not otherwise specified (DESNOS) in Croatian war veterans with posttraumatic stress disorder: case-control study

**DOI:** 10.3325/cmj.2011.52.505

**Published:** 2011-08

**Authors:** Iva Nemčić-Moro, Tanja Frančišković, Dolores Britvić, Miro Klarić, Iva Zečević

**Affiliations:** 1 Department of Psychological Medicine, University Hospital Center Zagreb, Zagreb, Croatia; 2 University of Rijeka, School of Medicine, Rijeka, Croatia; 3 Department of Psychiatry, University Hospital and School of Medicine, Split, Croatia; 4 Department of Psychiatry, Mostar University Hospital, Mostar, Bosnia and Herzegovina; 5 Psychiatric Hospital for Children and Youth, Zagreb, Croatia

## Abstract

**Aim:**

To determine the presence of disorder of extreme stress not otherwise specified (DESNOS) in Croatian war veterans who suffer from combat-related posttraumatic stress disorder (PTSD).

**Methods:**

The research included 247 veterans of the 1991-1995 war in Croatia who suffered from PTSD and were psychiatrically examined at four clinical centers in Croatia during a month in 2008. It was based on the following self-assessment instruments: The Harvard Trauma Questionnaire (HTQ): Croatian Version, the Structured Interview for Disorder of Extreme Stress (SIDES-SR), and the Mini International Neuropsychiatric Interview (MINI)

**Results:**

Based on the SIDES-SR results, we formed two groups of participants: the group with PTSD (N = 140) and the group with both PTSD and DESNOS (N = 107). Forty three percent of participants met the criteria for DESNOS. There was a significant difference in the intensity of posttraumatic symptoms between the group with both PTSD and DESNOS and the group with PTSD only (U = 3733.5, *P* = 0.001). Respondents who suffered from both PTSD and DESNOS also reported a significantly larger number of comorbid mental disorders (U = 1123.5, *P* = 0.049) and twice more frequently reported comorbid depression with melancholic features (OR = 2.109, *P* = 0.043), social phobia (OR = 2.137, *P* = 0.036), or panic disorder (OR = 2.208, *P* = 0.015).

**Conclusion:**

Our results demonstrate that PTSD and DESNOS can occur in comorbidity, which is in contrast with the ICD-10 criteria. A greater intensity of symptoms and a more frequent comorbidity with other psychiatric disorders, especially depression, panic disorder, and social phobia require additional therapy interventions in the treatment processes.

Posttraumatic stress disorder (PTSD) is a common, but not the only disorder that develops as an effect of a traumatic experience. The prevalence of PTSD in general population is 1 to 14% (1-4). In case of war veterans, the rate ranges from 15 to 57% (3,5,6). As much as 79% of persons suffering from PTSD suffer from another mental disorder and 44% suffer from three or more mental disorders. This means that in case of a large number of traumatized persons, PTSD diagnosis covers only a few mental disturbances (7-11).

Chronic or complex PTSD and DES or DESNOS (disorder of extreme stress not otherwise specified) have been investigated since the 1990s (12-15). DESNOS is defined and viewed as a group of symptoms within associated features of PTSD, and as such it may be included in future classifications (2,9). On the other hand, the ICD-10 introduced a diagnostic category of Enduring Personality Change After Catastrophic Events (F62.0), which includes features such as hostility and mistrustful attitude toward the world, social isolation, a feeling of emptiness and hopelessness, irritability, and estrangement. The diagnosis can be given only after two years of the illness duration and it excludes the diagnosis of concurrent PTSD because the enduring change is seen only as an adverse outcome of a long-lasting PTSD, not as a separate entity that can exist in comorbidity with PTSD (1). After its full development, enduring personality change cannot be treated that easily. The ICD-10 classification does not allow diagnosing the comorbidity of PTSD and Enduring Personality Change After Catastrophic Experience or DESNOS as a corresponding diagnostic category proposed by the Diagnostic and Statistical Manual of Mental Disorders (DSM)-IV classification (2).

The correlation of DESNOS and PTSD is still not completely clear. It is not specified whether DESNOS is a separate clinical entity or a complication of PTSD. Potential factors are severity of traumatic experience, correlation with the severity of PTSD, comorbidity in Axes I and II of the DSM-IV classification system, and a range of personal and social factors that add to the development of the disorder (9,16,17). In spite of clinical experience and results speaking in favor of the presence of the entity that can be developed as an effect of trauma independently of PTSD or in comorbidity with PTSD, the DSM IV classification has not included this category (2,16-19).

However, over the past 15 years a number of studies have discussed the possible inclusion of DESNOS in the DSM-V classification (16-19), many criteria have been defined, and even a specific questionnaire, Structured Interview for Disorder of Extreme Stress (SIDES), has been formed (9). Six clusters of symptoms have been proposed for establishing the DESNOS diagnosis: 1) alterations in regulation of affect and impulses (eg, extreme and unmodulated emotional states); 2) alterations in attention or consciousness (eg, dissociation); 3) alterations in self-perception (eg, image of self as fundamentally damaged); 4) alterations in interpersonal relations (eg, impaired relational boundaries); 5) alterations in biological self-regulation (eg, somatization), and 6) alterations in sustaining beliefs (eg, spiritual alienation) (18).[REMOVED HYPERLINK FIELD]

DESNOS mostly occurs as a separate disorder, but also in comorbidity with PTSD. The prevalence of DESNOS is 1% in female student population (19), 2% in civil population exposed to war (18), and up to 57% in war veterans, 31% in comorbidity with PTSD and 26% as a separate diagnostic category (16). Furthermore, the prevalence of PTSD is the same in the general population and treatment-seeking population (17). Nearly a half of the treatment-seeking population meets the criteria for DESNOS, which suggests that symptoms of DESNOS, more likely than symptoms of PTSD, are the ones prompting patients to seek help (17).

Clinical experience in working with war veterans in Croatia shows that there are patients suffering from chronic PTSD who report a range of symptoms meeting the criteria proposed for DESNOS (9). However, possible comorbidity of DESNOS and PTSD has not been assessed, probably to conform to the existing ICD classification (1). It may be assumed that DESNOS affects patients with severe clinical features of PTSD and that it also occurs in cases of Axis I comorbidity. These assumptions were the subject of our investigation.

## Participants and methods

### Participants

The research was conducted in the following centers: Department of Psychological Medicine, University Hospital Center in Zagreb; Psychotrauma Center of the Psychiatric Clinic of the University Hospital Centre in Rijeka; Psychotrauma Center of the Psychiatric Clinic in University Hospital Center in Split; and Sveti Ivan psychiatric hospital in Zagreb, Croatia. The research included consecutive patients, veterans of the 1991-1995 war in Croatia seeking psychiatric examination at these clinics during a month in 2008, regardless of whether they had been treated before or not. The inclusion criterion was PTSD that had developed during the war and the exclusion criterion was the presence of mental disturbances prior to the war. Of 480 patients who sought treatment during the observed period, 418 met the inclusion criteria. Sixteen patients had had mental disturbances before the war and 52 refused to participate. Three hundred and fifty four participants were included in the research, but 61 did not fill out the questionnaires completely and 46 participants no longer met the PTSD criteria according to the Harvard Trauma Questionnaire. The final sample consisted of 247 participants.

The research was conducted on a voluntary basis and all participants were informed about the aim of the research. Those who agreed to participate gave their written informed consent. The research was approved by the Ethics Committee of the University of Rijeka School of Medicine.

### Instruments

The Harvard Trauma Questionnaire (HTQ): Croatian Version (20) was used to determine PTSD and traumatization level. The HTQ is designed in a form of a structured interview, but in this research it was used as a self-assessment questionnaire. It consists of four subscales. The first (the list of possible traumatic events) contains questions on experiences and traumatic events having affected the residents of Croatia during the war, in the refugee period, and the postwar period. It includes 46 possible traumatic events presented in the form of “yes” and “no” questions. The second subscale is a personal description of the most traumatic event and the third refers to brain injuries. These two subscales are not designed for scoring. The fourth subscale consists of 40 statements referring to psychosocial difficulties caused by trauma. The first 16 statements are derived from the DSM-IV criteria for PTSD. These symptoms are grouped around three clusters of symptoms: re-experiencing the trauma, avoidance, and arousal symptoms. The rest of the statements refer to participants’ perception of the extent to which the trauma affected their everyday abilities. Answers to each question are scored as follows: 1 = not at all, 2 = very little, 3 = quite, 4 = very much. The total score is the mean value of all 40 statements. The cut-off score for PTSD is >2.5, ie, the mean value higher than 2.5 indicates the presence of PTSD. The score is comparable with the scores of patients clinically diagnosed with PTSD. The internal consistency of the instrument was high (Cronbach α = 0.88 for traumatic events, 0.95 for traumatic symptoms, 0.94 for symptoms of perceived functioning, and 0.97 for general score).

To determine the presence of DESNOS, we used the Structured Interview for Disorder of Extreme Stress (SIDES-SR). It is a self-report instrument and consists of 45 items divided into 6 subscales according to symptoms of DESNOS, with answers on a five-point Likert scale. The SIDES has good psychometric properties and it is the only validated instrument for establishing DESNOS diagnosis (21). It had a good internal consistency (Cronbach α = 0.96) (21) and a good construct validity (22).

Comorbidity was determined by Mini International Neuropsychiatric Interview (MINI). The MINI is designed as a short structured interview for major Axis-I disorders according to DSM-IV and ICD-10 classifications. It consists of subscales that correspond to a diagnostic category (23). There are two to four screening questions for each disorder. Additional questions about symptoms are asked if the answers to the screening questions are positive. MINI showed good assessment reliability, with kappa coefficients ranging from 0.88 and 1.0, and good test-retest reliability, with Cronbach α coefficients ranging from 0.76 and 0.93 (23). The MINI had good concordance with other diagnostic measures. Comparison of the MINI with the Structured Clinical Interview for DSM Disorders and the Composite International Diagnostic Interview shows that the MINI has a highly acceptable level of validity and reliability (24).

### Statistical analysis

Data were analyzed by using the SPSS (SPSS Inc., Chicago, IL, USA) for Windows. Basic descriptive parameters (arithmetic mean, standard deviation or nonparametric indicators) were calculated for all the measures used in the research. Differences between the groups were tested by the *t* test or the Mann-Whitney U-test for independent samples or χ^2^ test in case of nonparametric analysis. The level of significance was set at *P* < 0.05.

## Results

We formed two groups of participants based on the SIDES-SR results – those with PTSD only (n = 140) and those with both PTSD and DESNOS, ie, those who met all of the six criteria for establishing the DESNOS diagnosis (n = 107), which amounted to 43% of veterans.

We found no significant difference between the groups in any of the demographic variables (education, marital status, employment status, engagement in the war, and the presence of somatic diseases) (Table 1). There was also no significant difference between the groups in age (*t* = -0.16, *P* = 0.873) or time spent on the battlefield (U = 0.425, *P* = 0.671) (Table 2).

**Table 1:** Demographic characteristics of posttraumatic stress disorder (PTSD)-affected participants with and without disorder of extreme stress not otherwise specified (DESNOS)

		No. (%) of patients with		
		PTSD and DESNOS (n = 107)	PTSD only (n = 140)	*P* (χ^2^ test)
Education	elementary	15 (14.3)	19 (13.7)	0.248
	secondary	86 (81.9)	108 (77.7)	
	higher education	4 (3.8)	7 (5.0)	
	bachelor's or master's degree	0 (0)	5 (3.6)	
Employment status	retired	32 (41.0)	57 (50.4)	0.589
	receiving welfare benefits	4 (5.1)	4 (3.5)	
	employed	29 (37.2)	38 (33.6)	
	unemployed	13 (16.7)	14 (12.4)	
Marital status	single	8 (10.1)	9 (7.8)	0.459
	cohabiting	4 (5.1)	7 (6.1)	
	married	61 (77.2)	96 (83.5)	
	divorced	5 (6.3)	3 (2.6)	
Engagement in the war	volunteer	65 (83.3)	91 (79.8)	0.458
	mobilized	6 (7.7)	15 (13.2)	
	police	7 (9.0)	8 (7.0)	
Presence of a physical disease	yes	51 (68.9)	67 (60.9)	0.278
	no	23 (31.1)	43 (39.1)	

**Table 2:** Age and time spent on the battlefield in posttraumatic stress disorder (PTSD)-affected veterans with and without disorder of extreme stress not otherwise specified (DESNOS)

	No. of patients with					
	PTSP and DESNOS		PTSP only		*t* test; U*	*P*
Age, mean ± standard deviation (years)	106	45.29 ± 7.30	128	45.45 ± 7.22	-0.16	0.873
Time (months) on battlefront, median (range)	74	37 (5-103)	107	38 (1-96)	0.425	0.671

*Mann-Whitney U test.

We used the results from the first 16 statements of the HTQ subscale referring to trauma symptoms (the statements were recorded based on the HTQ user manual) (20) as a measure of the intensity of posttraumatic symptoms. The overall result was presented in one of the three categories: does not have PTSD (0-2.5), has PTSD (2.5-3.3), and has a severe PTSD (3.3-4).

General result on the MINI, ie, the number of other mental diseases occurring with PTSD (the PTSD diagnosis was not included) was used as a measure of comorbidity. There was no difference in the number of experienced traumatic events between the group with both DESNOS and PTSD and the group with PTSD only. However, participants with both PTSD and DESNOS reported a significantly greater intensity of trauma symptoms (U = 3733.5, *P* = 0.000).

Participants with DESNOS had significantly more mental disorders in comorbidity with PTSD (U = 1123.5, *P* = 0.049). (Table 3). They suffered from one of the following comorbid disorders twice more frequently than participants with PTSD only: depression with melancholic features (odds ratio [OR] = 2.109, *P* = 0.043), social phobia (OR = 2.137, *P* = 0.036), or panic disorder (OR = 2.208, *P* = 0.015). They also suffered three times more frequently from acute panic disorder (OR = 2.802, *P* = 0.011), (Table 4).

**Table 3:** Differences in the number of traumatic experiences, intensity of posttraumatic stress disorder (PTSD) symptoms, and comorbidity between the group with both PTSD and disorder of extreme stress not otherwise specified (DESNOS) and the group with PTSD only

Variable	Group	N	Median (range)	U*	*P*
Traumatic experience	PTSD and DESNOS	107	20 (5-36)	6842.5	0.244
	PTSD only	140	20 (8-34)		
Intensity of PTSD	PTSP and DESNOS	96	121 (83-157)	3733.5	0.000
	PTSP only	120	109 (78-196)		
Comorbidity	PTSP and DESNOS	45	4 (1-12)	1123.5	0.049
	PTSP only	64	3 (1-11)		

*Mann-Whitney U test.

**Table 4:** Differences in comorbidity on Axis I between the group with both posttraumatic stress disorder (PTSD) and disorder of extreme stress not otherwise specified (DESNOS) and the group with PTSD only

Diagnosis	PTSD and DESNOS, yes/no	PTSD only, yes/no	Odds ratio (95% confidence interval)
Current depression	29/35	29/58	2.429 (1.270-4.642)
Recurrent depression	28/37	32/55	1.301 (0.675-2.508)
Depression with melancholic features	20/43	15/68	2.109 (0.976-4.557)*
Dysthymia	19/42	22/62	1.275 (0.616-2.641)
Suicidality	22/41	29/56	1.036 (0.522-2.056)
Manic episode	3/59	0/84	
Current hypomanic episode	1/60	1/85	1.417 (0.087-23.099)
Past hypomanic episode	4/57	4/81	1.421 (0.3412-5.919)
Current panic disorder	19/45	11/73	2.802 (1.221-6.428)*
Lifetime panic disorder	32/34	26/61	2.208 (1.134-4.299)*
Agoraphobia	18/45	15/71	1.893 (0.868-4.132)
Social phobia	21/43	16/70	2.137 (1.006-4.538)*
Obsessive–compulsive disorder	4/59	3/82	1.853 (0.399-8.592)
Alcoholism	4/60	1/86	5.733 (0.625-52.578)
Alcohol abuse	12/52	18/73	0.936 (0.415-2.109)
Current psychosis	0/64	3/82	
Past psychosis	1/63	1/84	1.333 (0.082-21.730)
Current mood disorder	3/60	2/85	2.125 (0.345-13.109)
Generalized anxiety disorder	11/53	19/67	0.732 (0.321-1.671)
Somatization disorder	5/59	9/81	0.763 (0.243-2.393)
Current somatization disorder	8/55	8/78	1.418 (0.502-4.008)

*Significant at *P* < 0.05.

## Discussion

Our research showed that DESNOS affected Croatian war veterans in comorbidity with PTSD (43%). This finding is not in accordance with diagnostic criteria from the existing ICD-10 classification system. Symptoms of PTSD do not disappear after the development of permanent effects of psychotraumatization as the ICD-10 classification of diseases implies (1).

Ford and Kidd showed that the prevalence of DESNOS in PTSD-affected veterans was 57% (23). Ford also found that 31% of PTSD-affected veterans seeking hospital treatment met the criteria for both PTSD and DESNOS (16).

Our research demonstrated that participants with both PTSD and DESNOS reported a greater intensity of PTSD symptoms. Ford et al associated DESNOS with extreme levels of intrusive flashbacks and intensive use of psychiatric care (16). Zlotnick also found a positive correlation between PTSD intensity and meeting the DESNOS criteria (15).

Our research did not reveal any difference between the group with PTSD only and group with PTSD and DESNOS in the level of traumatization. This finding differs from previous studies (25), which indicate that traumatization is stronger in persons with DESNOS. Furthermore, other studies suggest that current traumatization is not the sole factor in DESNOS development and that early traumatic experiences play a role. We may have not found any difference in the level of traumatization because, due to difficulties in defining the frequency and intensity of traumatic experience in cases of multiple traumatic experiences, we measured it by the number of traumatic events experienced by a person. The war veterans went through an average of 14 traumatic events, but the measuring instrument could not determine the actual intensity of stressors and their effects.

Our patients with both PTSD and DESNOS had more comorbid mental disorders. The war veterans with DESNOS significantly more frequently suffered from depression, panic disorder, and social phobia than war veterans without DESNOS.

Other studies also found depression in comorbidity with DESNOS (16,22,26). It is usually present in nearly a half of patients suffering from PTSD (10,27-30), but it seems that the combination of the two disorders largely contributes to the development of DESNOS. Our research also revealed a significant comorbidity of DESNOS and social phobia. According to literature, PTSD is caused by a non-interpersonal trauma (eg, earthquake), while DESNOS is characterized by an interpersonal trauma where another person acts as the instigator of trauma (19). Many studies indicate that patients who developed PTSD after an interpersonal trauma (as rape or captivity) have more PTSD symptoms (31,32). Moreover, comorbidity of DESNOS and social phobia may be explained on a symptomatic level. One of the symptoms of persons diagnosed with PTSD is avoidance of trauma triggers, which often means avoidance of activities, places, people, and situations that arouse recollections of the trauma (2). If a person suffers both from PTSD and DESNOS, chances of overlapping of the symptoms with symptoms of social phobia are greater. Two clusters of criteria for DESNOS diagnosis can be associated with social phobia: self-perception disorder or alterations in the self-perception and alterations in relations to other people. This includes a feeling of being different and estranged from others, avoidance of other people, the feeling of shame in front of other people, as well as the feeling of distrust (5). Persons suffering from social phobia feel a constant, irrational fear related to the presence of other people and tend to avoid every situation in which they could be assessed or evaluated (5). Persons with DESNOS, by avoiding other people further impair their abilities to function since they isolate themselves, lose social support, and enjoy fewer pleasant activities, which puts them at risk of developing depression. This may be the reason of frequent comorbidity of depression and DESNOS.

Clusters of criteria for DESNOS diagnosis that can be correlated with the symptoms of panic disorder include alterations in consciousness such as dissociation, which is common for panic disorder, and alterations in biological self-regulation, which in case of panic disorder can refer to intensified perception of physical symptoms of anxiety.

Recognizing DESNOS in traumatized groups is important for conducting therapeutic interventions. The treatment of PTSD is entirely successful in only 30% of the patients; partially successful in other 30%, while the rest of the patients do not show any treatment results (33). We presume that the last group consists of patients who suffer from both PTSD and DESNOS. Several studies have shown that DESNOS is a powerful negative prognostic factor for treatment outcome (15,25)

The disparity between the existing treatment research samples and actual clinical populations may be the reason why many clinicians treating patients with complex presentations continue to follow the treatment models that are based on clinical experience rather than on empirical research (34). Treatment of PTSD focuses on the processing of specific traumatic experiences and memories. In contrast, in t patients with DESNOS, the emphasis should be placed on treating other problems, such as the loss of emotion regulation, dissociation, and interpersonal problems, probably because they cause a more severe functional impairment than PTSD symptoms (17,34)

Consequently, clinicians have learned to pay more attention to issues of patient safety, affect regulation, coping and self-management skills, as well as to the therapeutic relationship itself, rather than to focus on the processing of traumatic memories. At present, the clinical consensus model for the treatment of patients with complex trauma histories includes three primary stages: a) symptom reduction and stabilization, b) processing of traumatic memories and emotions, and c) life integration and rehabilitation after trauma processing (34)

The best insights into trauma effects could be given by longitudinal studies that would begin before the trauma takes place and end after the trauma is over (for example, studies on the population of professional soldiers, security guards, or the police).

According to our study, the SIDES fulfilled its purpose and can be used in assessing DESNOS in other populations as well. However, it is necessary to conduct more research that would confirm our presumptions.

This research has several limitations. The sample included treatment-seeking population that usually shows a larger prevalence of DESNOS (17). Further on, the veterans might have overemphasized the symptoms for the sake of receiving social security benefits and traumatic experiences were gathered by using self-report instruments without an external confirmation, and self-report instruments always open up a possibility for aggravating the pathology. In addition, the research did not include a control group, which would provide information on the presence of DESNOS in the general population and veteran population without PTSD. The inclusion criterion was PTSD, which meant that persons who did not meet the PTSD criteria but who might have met the DESNOS criteria, were not included. Due to the research locations, the veteran sample mostly covered urban population and was not representative of the entire Croatia. The research also excluded hospital population of veterans and population of veterans with prior mental disturbances. As for methodological limitations, the Axis-I diagnoses were made by authors trained for the application of the MINI, but due to distance between the cities where the research took place, retesting was not performed and inter-rater reliability was not established.

As a conclusion, we may assume that DESNOS affects Croatian war veterans in comorbidity with PTSD, but the symptoms of the two disorders are not mutually exclusive as suggested by the ICD-10. DESNOS shows a greater intensity of symptoms and a more frequent comorbidity with other psychiatric disorders, especially depression, panic disorder, and social phobia. Owing to the severity of clinical features and the frequency of comorbid disorders, it is important that clinicians extend their focus to symptoms of DESNOS when planning treatment.
